# An 8-year-old-girl with juvenile dermatomyositis and autoimmune thyroiditis in Tanzania: a case report

**DOI:** 10.1186/s13256-021-03222-5

**Published:** 2021-12-27

**Authors:** Fatima Mussa, Neema Nalitolela, Francis Fredrick

**Affiliations:** Department of Paediatrics and Child Health, School of Medicine, Muhimbili University of Health and Allied Sciences, 9 United Nations Road, Upanga West, P.O BOX 65001, Dar es Salaam, Tanzania

**Keywords:** Juvenile dermatomyositis, Autoimmune thyroiditis, Inflammatory myopathy, Tanzania

## Abstract

**Background:**

Juvenile dermatomyositis is an inflammatory disease of muscles, skin, and blood vessels of unknown cause affecting all age and ethnic groups, with a reported incidence of 1.9–4.1 per million. It manifests with weakness in axial and proximal muscles and typical skin lesions. Historically, the Bohan and Peter classification schema has been used to diagnose juvenile dermatomyositis.

**Case presentation:**

We report an 8-year-old African female child, who presented with features of juvenile dermatomyositis and a rare association with subclinical autoimmune thyroiditis. This case illustrates the typical presentation, diagnosis, and treatment outcomes of this highly misdiagnosed condition.

**Conclusion:**

Due to the limited resources and knowledge about this under-reported disease in resource-constrained settings, the characteristic manifestations of juvenile dermatomyositis can be easily missed and thus requires a high index of suspicion for earlier diagnosis and management.

## Background

Juvenile dermatomyositis (JDM) is a rare autoimmune myopathy in childhood. It is primarily a capillary vasculopathy affecting the skin and skeletal muscles. The definitive cause of JDM is unclear, however it has been proposed that JDM is caused by an autoimmune reaction in a genetically susceptible individual, possibly in response to infection such as Group A streptococcal infection, specifically *Streptococcus pyogenes*, or environmental triggers. One of the proposed immunological mechanisms is the molecular mimicry with the bacterium *S. pyogenes* and the epitope of human skeletal muscles.

JDM is the most common idiopathic inflammatory myopathy of childhood, accounting for approximately 85% of cases [1, 2]. In population-based studies, JDM has a reported annual incidence of 2–4 cases per 1 million children [3–8].

The peak incidence is from 5 to 10 years of age [6, 8–10]. Girls are affected two- to fivefold more often than boys [8, 10, 11]. The presence of muscular weakness with characteristic cutaneous rash in a child should raise a high index of suspicion for JDM. Steroids maybe an adjunct treatment to inflammatory dermatoses [12, 13]. We report a case of an 8-year-old girl with JDM and a rare association with autoimmune thyroiditis.

## Case presentation

An 8–year–old African girl presented to Muhimbili National Hospital, Dar es salam, Tanzania, with an 8 week history of recurrent fever, followed by a skin rash, weakness of all limbs, and generalized body swelling 2 weeks later. The fever was of low grade, accompanied by a sore throat, and was treated with antibiotics and antimalarial but it recurred with no specific periodicity throughout the course of her illness. The rash was maculopapular and itchy and started on the upper extremities (arms, hands and predominantly on the knuckles) and progressed to involve the thighs. This lasted for 2 weeks and persisted in the knees as papules and painful ulcerations on the flexure areas of the limbs. She developed a gradual onset of limb weakness, which was progressive and symmetrical, involving the proximal aspects of upper and lower limbs, and she was unable to comb her hair, climb stairs, and get up from squatting position. Weakness of limbs was accompanied by muscle and joint pain. She also noted swelling on the face, which was more prominent around the eyes. There was a reported nasal tone to her voice in the course of illness, but no history of dysphagia. There was no change in micturition habits. There was no history of night sweats, weight loss, or tuberculosis exposure. Otherwise she had a normal development and excellent school performance. She was the only child of nonconsanguineous parents. There was no family history of similar condition. She had already attended several hospitals, and been misdiagnosed and treated for various conditions with no improvement before coming to us.

**Fig. 1:**
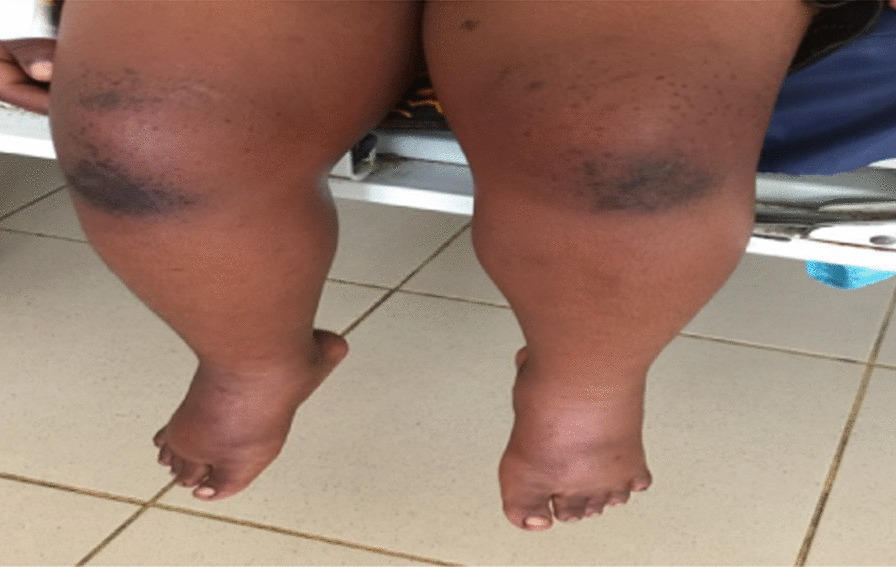
Lower limbs swelling with Gottron’s sign on both knees

**Fig. 2:**
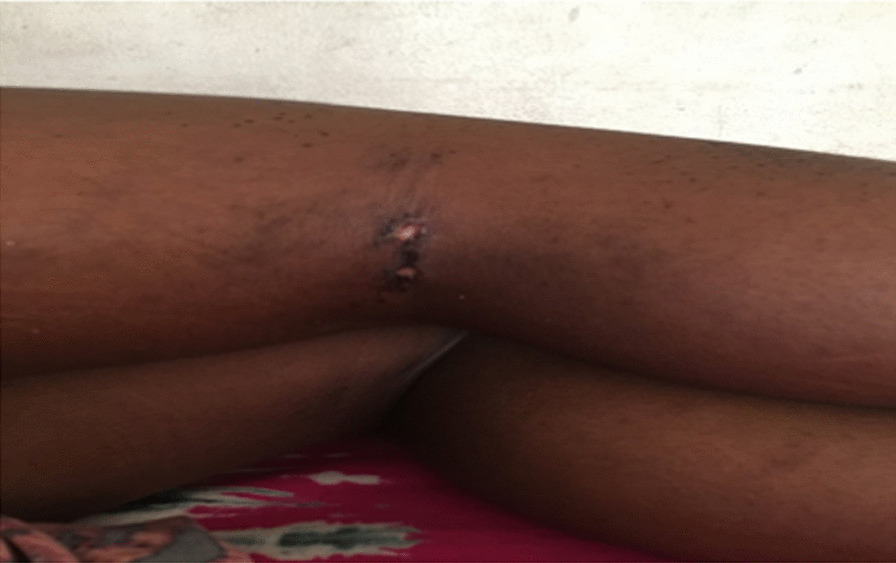
Ulcers with clear crusted margins on posterior aspects of both knees

On physical examination she was afebrile, in pain, with generalized edema (periorbital and bilateral nonpitting edema of upper and lower limbs). Her body mass index for age and sex was normal (16.3 kg/m^2^—median to + 1 SD). On local examination of the skin, she had hyperpigmented macules located on both knees, anteriorly more on the right, some coalesced to form patches on the knuckles (Gottron’s sign) (Fig. 1). There were also hyperpigmented patches and ulcers with clear crusted border margins measuring about 2 cm posterior to the knees and axillary folds (Fig. 2). She had a tiptoe gait with limited extension and flexion of the knees and ankle joint. Her grip power was normal with restricted movements of the elbows and shoulder joints. However, there was no joint swelling, warmth, or spine deformities noted. Her muscle bulk was normal but she had generalized tenderness in all muscle groups and a reduced power of grade 3/5 in proximal muscle groups of the upper and lower limbs with a positive Gowers’ sign. She had no calcinosis cutis. She scored 22/52 on Childhood Myositis Assessment Scale (CMAS-14).

**Fig. 3:**
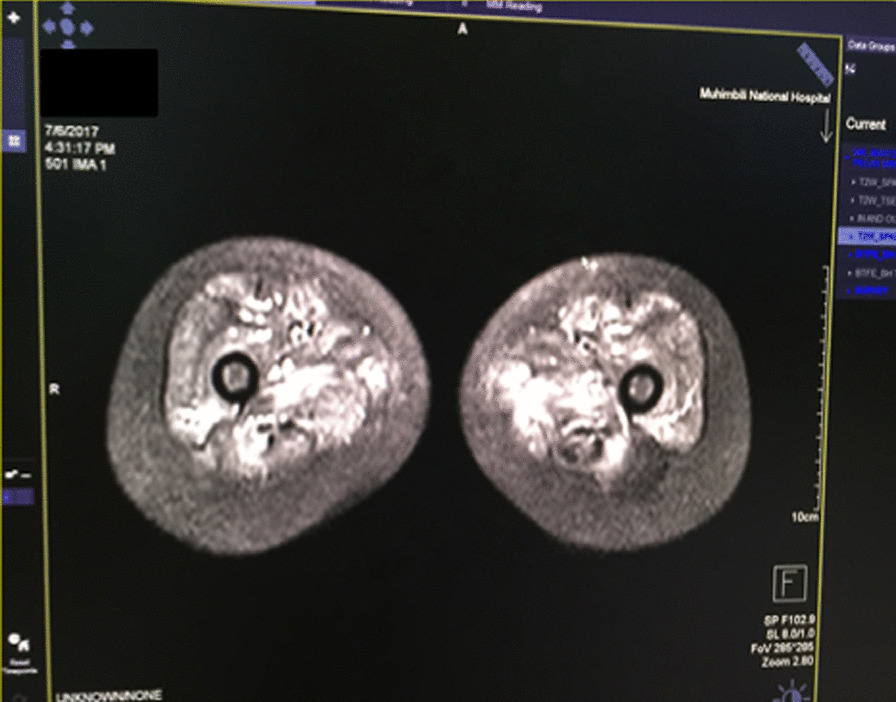
MRI of the proximal legs showing hyperintense signals

Laboratory investigations revealed elevated serum levels of creatine kinase, 1948 U/l (29–168 U/l), lactate dehydrogenase [LDH, 1057 U/l (125–220 U/l)], aspartate aminotransferase [AST, 159 U/l (5–34 U/l)], C-reactive protein [CRP, 58 mg/l (0–5 mg/l)], erythrocyte sedimentation rate [ESR, 56 mm/hour (3–13 mm/hour)], thyroid stimulating hormone [TSH, 7.56 μIU/mL (0.49–4.67 μIU/mL)], normal free triiodothyronine (FT3, 1.81 ng/dl), and free thyroxine (FT4, 1.25 ng/dl). Full blood count and renal function tests were normal, Hepatitis B surface antigen (HBs Ag), Hepatitis C virus antibody (HCV Ab) were both negative. Magnetic resonance imaging (MRI) of the proximal legs showed increased signal intensity in muscular compartments seen as bright spots, signifying edema of the thigh muscles (Fig. 3). X-rays of the limbs and echocardiogram were normal.

Based on the typical clinical features, laboratory and MRI findings, and subclinical hypothyroidism, the diagnosis of JDM with autoimmune thyroiditis was made.

The patient was treated with intravenous methylprednisolone at a dose of 30 mg/kg for 3 days followed by oral prednisolone at 60 mg/m^2^. An immediate dose of intravenous cyclophosphamide 500 mg/m^2^ was given, followed by weekly oral methotrexate 10 mg. Oral folic acid, calcium carbonate, omeprazole, and topical mupirocin were also given. The patient was initiated on daily physiotherapy.

Following treatment, the patient’s condition improved. Swelling and pain reduced first, subsequently weakness reduced, and she was able to walk longer distances, climb stairs with support, comb hair, and feed herself. Skin ulcers healed and she was able to extend her knees fully. However, the macules on the knee and ankle stiffness persisted.

She was discharged fairly well after 6 weeks to be followed-up in clinic and to continue treatment and physiotherapy.

At follow-up, on maintenance dose of 10 mg methotrexate weekly and daily folic acid at 5 mg/day, she fared well with gradual improvement in her muscle power, pain, and stiffness. She continued to have a tiptoe gait. She had good adherence to her treatment supported by her mother with no adverse effects to her regime.

## Discussion

JDM is a systemic, autoimmune inflammatory muscle disorder and vasculopathy that affects children younger than 18 years. It primarily affects the skin and skeletal muscles. Characteristic findings include Gottron papules, heliotrope rash, calcinosis cutis, and symmetrical proximal muscle weakness. Of these, our patient had Gottron’s sign, skin ulcerations, and proximal muscle weakness. In 1975, Bohan and Peter proposed a classification schema and diagnostic criteria for different forms of myositis, including JDM, which was based on the following five criteria: characteristic skin rash, proximal muscle weakness, elevated muscle enzymes, myopathic changes on electromyography, and abnormal muscle biopsy findings, making the diagnosis of JDM possible, probable, or definitive. However, recent studies show that MRI is a better modality of myositis diagnosis. In a study in the UK and Ireland in children with myositis, up to 76% of abnormalities consistent with myositis could be detected on MRI, and it was concluded that MRI has increased sensitivity and can be used to detect myositis [1]. In view of this, we used MRI as our modality of diagnosis in lieu of more invasive procedures such as muscle biopsy and electromyography as suggested by Bohan and Peter. We thus confirmed our diagnosis of JDM based on the typical clinical symptoms, elevated muscle enzymes, and typical MRI findings.

In addition to the routinely performed muscle enzyme tests, which we did for our patient, studies reveal myositis-specific autoantibodies [(MSAs) anti-Mi-2 and anti-p155/140] can be found in a small number of children with inflammatory myositis, which further confirm the diagnosis [14, 15]. In one study of 38 children with JDM and other connective tissue diseases, MSAs were identified in 12 of 77 serum samples, while in another study, anti-Mi-2 autoantibodies were found in only 2 of 42 patients with JDM [16]. However, its availability and cost limits its availability and use in our setting.

Muscle weakness and elevated creatinine kinase are well known to be accompanied by hypothyroidism. While in our patient, who had no overt symptoms of hypothyroidism, serum levels of TSH were found to be high with normal FT3 and FT4, which is in keeping with subclinical hypothyroidism. A rare association of JDM with subclinical hypothyroidism due to autoimmune thyroiditis has been documented in a Japanese case report of a 12-year-old girl presenting similarly with subclinical hypothyroidism in JDM and positive thyroid autoantibodies. Due to our limited resources, we were unable to do the thyroid autoantibodies test [17].

## Conclusion

The incidence of JDM in industrialized countries is reported to be 1.9–4.1 per million. To our knowledge, there is no incidence reported in Africa, however, there is one case report in Kilimanjaro Christian Medical Centre in Moshi, Tanzania [12]. Our case implicates the characteristic manifestations of JDM can be clearly misdiagnosed and, thus, under reported. We should have a high index of suspicion and use the clinical and investigative modalities to make a diagnosis of JDM.

## Data Availability

Not applicable.
